# Morphological Evolution of Coexisting Amphipod Species Pairs from Sulfidic Caves Suggests Competitive Interactions and Character Displacement, but No Environmental Filtering and Convergence

**DOI:** 10.1371/journal.pone.0123535

**Published:** 2015-04-23

**Authors:** Cene Fišer, Roman Luštrik, Serban Sarbu, Jean-François Flot, Peter Trontelj

**Affiliations:** 1 Department of Biology, Biotechnical Faculty, University of Ljubljana, 1000 Ljubljana, Slovenia; 2 Grupul de Explorări Subacvatice şi Speologice, Strada Frumoasă 31, 010986 Bucureşti, Romania; 3 Department of Genetics, Evolution and Environment, University College London, Darwin Building, Gower Street, London WC1E 6BT, United Kingdom; 4 Museum für Naturkunde, Leibniz-Institut für Evolutions- und Biodiversitätsforschung an der Humboldt-Universität zu Berlin, Invalidenstrasse 43, Berlin 10115, Germany; CNRS, FRANCE

## Abstract

Phenotypically similar species coexisting in extreme environments like sulfidic water are subject to two opposing eco-evolutionary processes: those favoring similarity of environment-specific traits, and those promoting differences of traits related to resource use. The former group of processes includes ecological filtering and convergent or parallel evolution, the latter competitive exclusion, character displacement and divergent evolution. We used a unique eco-evolutionary study system composed of two independent pairs of coexisting amphipod species (genus *Niphargus*) from the sulfidic caves Movile in Romania and Frasassi in Italy to study the relative contribution and interaction of both processes. We looked at the shape of the multifunctional ventral channel as a trait ostensibly related to oxygenation and sulfide detoxification, and at body size as a resource-related trait. Phylogenetic analysis suggests that the sulfidic caves were colonized separately by ancestors of each species. Species within pairs were more dissimilar in their morphology than expected according to a null model based on regional species pool. This might indicate competitive interactions shaping the morphology of these amphipod species. Moreover, our results suggest that the shape of the ventral channel is not subject to long-term convergent selection or to the process of environmental filtering, and as such probably does not play a role in sulfide tolerance. Nevertheless, the ancestral conditions reconstructed using the comparative method tended to be more similar than null-model expectations. This shift in patterns may reflect a temporal hierarchy of eco-evolutionary processes, in which initial environmental filtering became later on superseded by character displacement or other competition-driven divergent evolutionary processes.

## Introduction

Species sharing specific environments tend to resemble each other in traits that are adaptive in those environments [1–2]. Such trait similarities can be the consequence of environmental filtering of a few species from a larger regional pool or can be acquired through convergent evolution after the species enter their new environment [3]. The more ecologically distinct a certain environment is from its surroundings, the more conspicuous similarities can be expected among co-occurring species. In extreme environments such as caves, even members of different phyla attain striking similarity [4]. On rare occasions, such extreme environments might get colonized repeatedly by closely related species. Consequently, resources become depleted and interspecific competition may become an important source of selection [5–6].

As a rule, competition-based selection is minimized if species exploit distinct ecological niches [7]. The result of competitive interactions is limiting similarity among coexisting species [8–10]. Hence, species coexisting in specific local environments are subject to two opposing processes: ecological filtering that promotes similarity, and competition that promotes differentiation [11–12].

If coexistence persists through time, the conflict between the two processes has probably been resolved. This may had happened already during the assembly of the community by ecological filtering, if the colonizing species possessed exaptations allowing them cope with the new environment and at the same time to minimize competition for resources [3]. Alternatively, colonizers with insufficient exaptations may have undergone evolutionary change under the new selective environment and/or character displacement induced by interspecific competition [13–14]. These theoretical considerations raise two questions: first, can we detect signatures of ecological filtering and interspecific competition in coexisting species? And second, can we infer whether colonization happened with or without evolutionary change?

We address both questions using coexisting pairs of amphipods from sulfidic caves. The studied amphipods belong to the genus *Niphargus*, the members of which live in caves and other subterranean waters [15]. Although subterranean environments can generally be considered extreme on their own account, we focus here on caves characterized by a further extreme environmental parameter, namely high concentrations of hydrogen sulfide (H_2_S). Water with high concentrations of H_2_S is toxic: sulfide binds to cytochrome *c* oxidase and inhibits mitochondrial electron transport [16]. Only few species are able to colonize such environments. The strength of sulfide-based environmental filtering is best illustrated by Mexican fishes, where among 21 species present in the region only a single species, the cave molly (*Poecilla mexicana*), successfully colonized sulfidic water [17]. In addition to this system, *P*. *mexicana* independently colonized several other sulfidic localities, and the resulting populations evolved convergent morphological and behavioral features that help them cope with toxic H_2_S [18–20].

*Niphargus* species repeatedly colonized two sulfidic caves in Europe, Movile Cave in Romania, and the Frasassi cave system in Italy [21–22]. Here we used a recently developed ecomorphological framework for *Niphargus* [23–24] to test whether processes such ecological filtering and interspecific competition have affected functional morphological traits that are likely to relate to different dimensions of the ecological niche. We predict that coexisting species will (1) resemble each other in traits that reduce the toxic effect of sulfide, and (2) differ in traits that correlate functionally to resource use. Furthermore, we reconstructed the morphologies of the inferred ancestors of coexisting species pairs in order to check whether functional morphological traits evolved over time.

## Materials and Methods

### Study caves and study species

**Fig 1:**
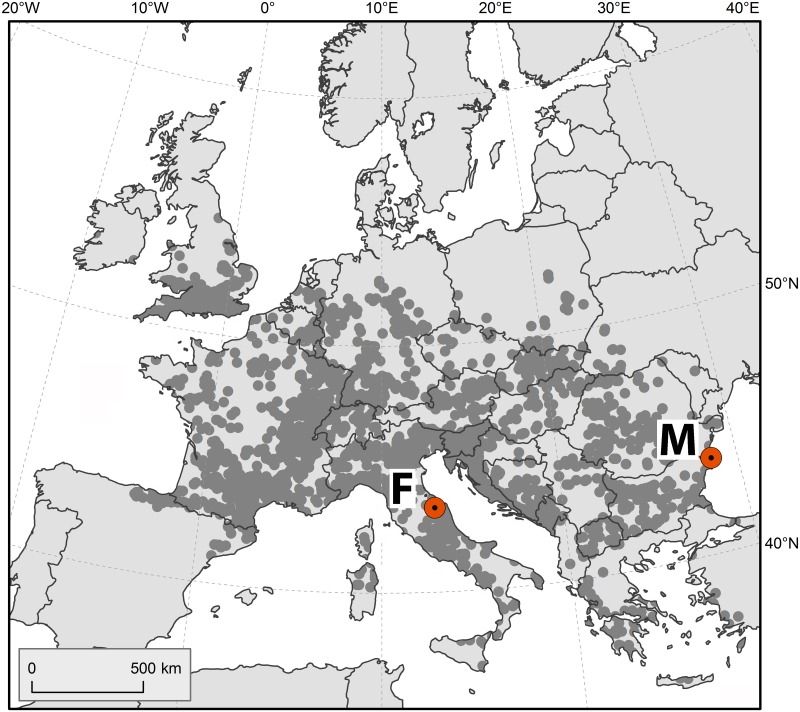
Geographic position of the studied sulfidic caves (F = Frasassi cave system, M = Movile Cave). Shaded dots indicate the distributional range of *Niphargus* in Europe, after [61].

Movile Cave is situated in southeastern Romania on the Dobrogea Plateau (Fig 1). The surrounding of the cave is ecologically homogenous and covered with steppe vegetation. The fauna of the cave was extensively studied and includes 18 aquatic species. The macrofauna consist of two amphipods, an isopod, and two possible predators—a leech and a water bug [21]. Our two focal species are *Pontoniphargus racovitzai* Dancău 1970 and a yet undescribed species, provisionally named as *Niphargus* cf. *stygius*; both are endemic to the Movile Cave aquifer and apparently inhabit sulfidic water as their primary habitat [25]. *Pontoniphargus* species consistently appears nested in the genus *Niphargus* on molecular phylogenies (see [25] and the Results section of this paper), and its genus status is under revision (unpublished). Even though south-eastern Romania is poorly explored, the vicinity of Movile Cave up to a radius of 24 km has been a subject of thorough sampling. Apart from our focal species pair, five other *Niphargus* species have been reported from this area making up a regional species pool. *Pontoniphargus ruffoi* Karaman & Sarbu 1993 is another species thriving exclusively in sulfidic water but restricted to a spatially separated sulfidic aquifer. Three further species (*N*. *hrabei* Karaman 1932, *N*. *gallicus* Schellenberg 1935, *N*. *dobrogicus* Dancău 1964) are apparently restricted to sulfide-free waters. Finally, *N*. *decui* Karaman & Sarbu 1995 is widely distributed in Dobrogea, mostly in sulfide-free water, but on rare occasions it was found in sulfidic waters as well.

The Frasassi cave system is located in central Italy, on the Adriatic side of the Apennine Mountains, about 40 km from the coast (Fig 1). This cave system lies in a geographically and geologically diverse area alongside a 500-meter deep and two-kilometer long canyon formed by the Sentino River. The surface surroundings are covered by thermophilous Mediterranean vegetation, pine forests and deciduous forests [22]. Although the fauna of this cave system has been less extensively studied than in Movile, *Niphargus* amphipods are among the largest aquatic animals there. Out of the four known species, one (*Niphargus* sp.4 in [26]) was collected only once and can be considered as an accidental visitor, whereas the other three are regular inhabitants of the cave system. The focal species, *N*. *ictus* Karaman 1985 and *N*. *frasassianus* Karaman, Borowski and Dattagupta 2010, dwell primarily in sulfidic water throughout the system and coexist in some places at a distance of a few centimeters [26]. The third local species, *Niphargus montanarius* Karaman, Borowski and Dattagupta 2010, has been found exclusively in one sulfide-free pool. The area surrounding this cave system is ecologically more heterogeneous than the Movile area, and the regional amphipod species pool is less known. To achieve robust estimations of the regional species pool despite this limitation, we considered for the analyses all *Niphargus* species within a range three times larger than for Movile Cave (i.e., within a radius of about 75 km); according to [27] these species are *N*. *aquilex* Schiödte 1855, *N*. *elegans* Garbini 1894, *N*. *longicaudatus* Costa 1851, *N*. *pasquinii* Vigna-Taglianti 1966, *N*. *orcinus* agg., *N*. *spoeckeri sibillinanus* Karaman 1984, *N*. *stefanellii* Ruffo & Vigna Taglianti 1967.

### Samples for phylogenetic and morphometric analyses

The comparative analysis of morphological evolution of sulfide-dwelling *Niphargus* species requires a wider phylogenetic context. For this purpose, we selected a subset of a large *Niphargus* DNA dataset [23–26, 28–29] that includes representatives of all major lineages, and all close relatives of our focal species. Altogether 44 *Niphargus* species were used to reconstruct phylogenetic relationships and ancestral morphologies. Lists of species, sampling sites, sequence accession numbers, and morphometric data are presented in the S1 and S2 Tables). No specific permissions were required for these locations and the study does not include endangered or protected species.

### Phylogenetic reconstruction

We used two variable sections of the 28S rRNA gene adding up to approximately 2100 bp, and a 330-bp section of the histone H3 gene. Primers and PCR protocols were as in [23–24]. The 28S rDNA sequences were aligned using MAFFT under the E-INS-i model (allowing for large indels separating conserved blocks [30]). Gap-rich regions were removed from the alignment prior to phylogenetic analysis with the help of Gblocks [31]. For the H3 sequences, no special alignment procedure was required as they were all of equal length.

A concatenated matrix of the three sequence alignments was used for subsequent Bayesian MCMC tree searches in MrBayes 3.2.1. [32]. Model parameters were set to six different substitution rate categories and gamma-distributed rate heterogeneity with a proportion of invariable sites, and optimized during the search. All parameters except the topology were unlinked between the 28S and H3 partition. Two independent runs with four chains each were sampled every 1000 generations until the standard deviation of split frequencies dropped below 0.002, which happened after roughly 20 million generations. The first 5000 trees from each run were discarded, and from the remaining 30,000 trees a 50% majority rule consensus was calculated.

### Morphological analysis

We measured two functional traits: the body size and the shape of the ventral channel. Each trait was used as a proxy for an independent ecological niche axis. The landmarks we used are described in [33]. We assumed that body size represents the trophic niche, and as such evolves divergently under competition for food resources (e.g., [10]). We measured size of adult animals.

The second trait, the shape of the so-called ventral channel, describes the hydrodynamic flow of oxygenated water to the amphipod gills (cf. [24]). Amphipods are laterally flattened: their ventrally elongated coxal plates, together with the bases of their pereopods, form a multifunctional channel along the ventral side of their body. The broom-shaped appendages in the posterior part of their body generate water currents that flow across the gills in the ventral channel. This water flow may also be used for filter-feeding and jet propulsion [34]. The deeper and the more closed the channel is, the stronger is the water current that delivers oxygen to the gills. Adaptations to hypoxia (as is often the case in sulfidic waters) frequently include structures that enhance oxygenation and gas exchange [19]. We therefore expected the shape of the ventral channel to be subject to environmental filtering and/or undergo convergent evolution in sulfidic caves. We estimated the efficiency of the ventral channel in respect to water flow by measuring the ventro-distal length of coxal plates II and III as well as the width of the bases of pereopods V and VII [23]. Pereopods VI were found to be frequently damaged and were therefore excluded from our analyses. Measurements in millimeters were regressed onto species body lengths and expressed as standardized residuals. All analyses of morphometric data are based on species mean values.

### Testing ecological predictions

If a particular trait has been subject to ecological filtering during colonization of a sulfidic cave, that trait is expected to be more similar between colonizing species than between species selected at random from non-sulfidic species in the corresponding regional pool. Conversely, if differences between species from the sulfidic cave exceed those observed in the regional species pool, that trait was probably affected by competition.

For each of the two caves, we first conducted a survey of faunistic data to establish the regional pool of *Niphargus* species living in non-sulfidic subterranean waters in the vicinity of both caves (see above and S3 Table). These species were measured for the same traits as described above. As we could not collect all species in nearby localities, we also used specimens from more distant populations. Unclear taxonomic records, e.g., “*Niphargus orcinus* aggregate” was represented by data obtained from the actual *N*. *orcinus* Joseph 1869.

Since the regional species pools comprised only four species for Movile and seven species for Frasassi, they were too small to allow for the generation of a statistically meaningful number of random species pairs. Increasing the number of species by enlarging the geographic range around each cave would have lead to the inclusion of species from other environments such as alluvial plains of Northern Italy and the Dinaric Karst, where they experience completely different ecological conditions such as space limitation and predation by cave salamander. Besides, natural dispersal from these areas to the studied sulfidic caves is highly unlikely. Hence, in order to avoid distorted and biased approximations of species pools [35] we resorted to a simulation approach. Under the assumption that the morphological variability of all known regional species represents the entire range of available ecological space, we simulated two regional pools of 100 virtual species each as follows. Each virtual species was defined by the five morphometric traits presented above. The values for simulated traits were drawn from a uniform distribution between the minimum and maximum value obtained from the actual species pool (as in the similar univariate approach used in [36]), thereby simulating two regional pools of 100 virtual species each. From each pool 1000 pairs of virtual species were drawn at random. These species pairs were then used to calculate Euclidean distances for body size and ventral channel shape, the distributions of which served as null models for comparison with our focal species pairs. All analyses were made using the R language [37]. The scripts used are available upon request.

### Inferring evolution

In order to assess whether evolutionary change contributed to above-random similarity or differences between coexisting *Niphargus* species, we estimated the trait values of their inferred ancestors. The assumption needed for this assessment to be valid is that the last common ancestor of a sulfidic species and its non-sulfidic sister species had lived in non-sulfidic water. The accuracy of ancestral state reconstruction is known to decrease when moving up the phylogenetic hierarchy of nodes [38–39], making such conjectures unreliable. In the present study, we minimized this problem by restricting the reconstruction of ancestral morphologies to the most recent common ancestors of sulfidic/non-sulfidic sister pairs, i.e. the first hierarchical level only. All calculations were made using COMPARE 4.6b [40]. Based on the Akaike information criterion, we found that the Ornstein-Uhlenbeck (OU) model of evolution outperformed the Brownian motion model and therefore conducted all our analyses under the OU model. The reconstructed ancestral species pairs were then tested for evidence of filtering and/or competition by comparing their divergence to null-model expectations as described above.

## Results

**Fig 2:**
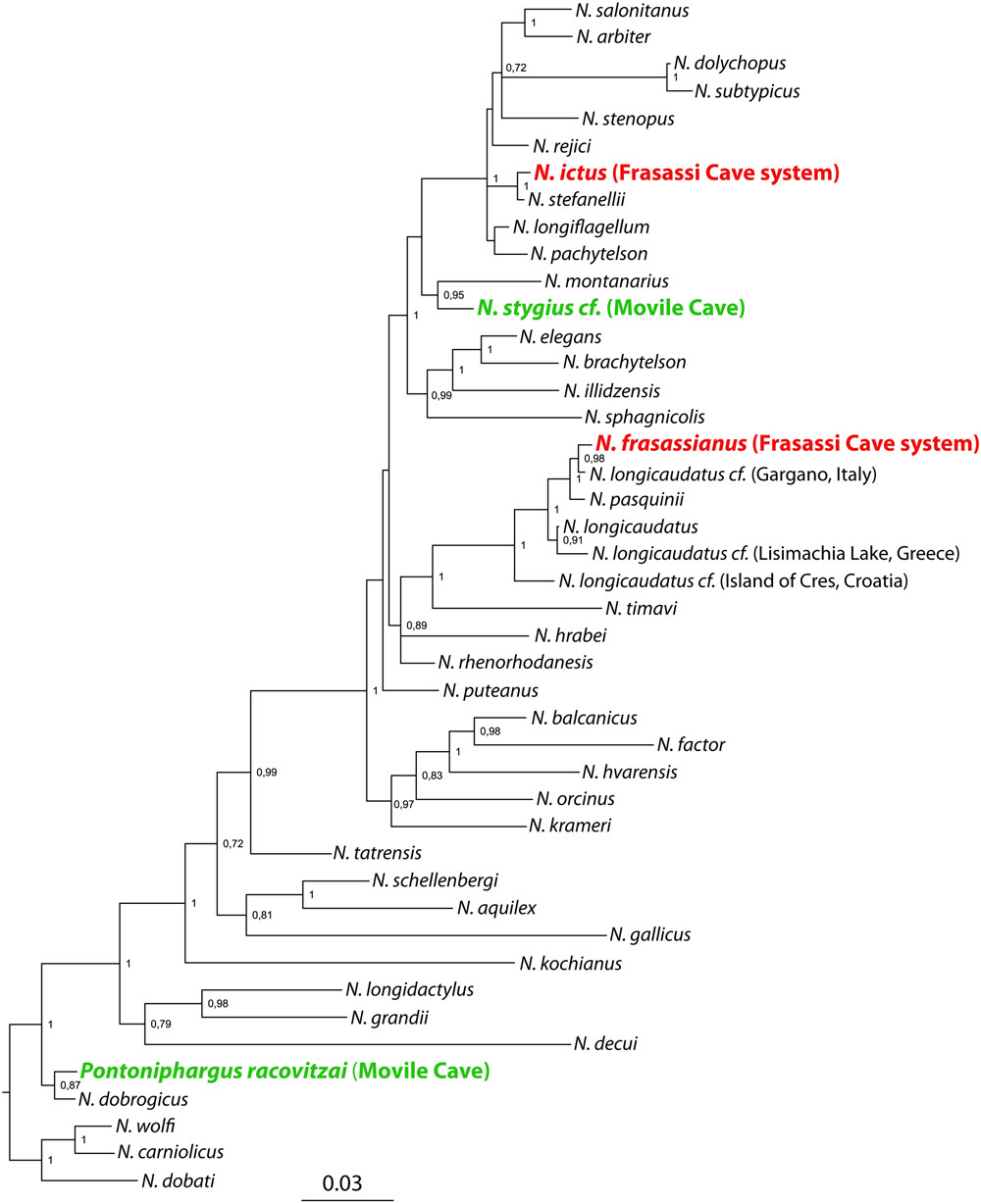
Phylogenetic relationships among 44 species of *Niphargus* obtained by Bayesian inference. The two studied species pairs from sulfidic caves are indicated in bold. Numbers on the nodes indicate Bayesian posterior probabilities; only nodes with support > 0.7 are indicated. Distinct yet undescribed species from the ‘longicaudatus’ group are labeled geographically. The tree was rooted using topological information from a genus-wide molecular phylogeny [28].

Our molecular phylogenetic analysis demonstrates that the four focal species living in sulfidic water belong to different clades, speaking in favor of four independent colonizations of the sulfidic caves (Fig 2). Their extant sister species live in sulfide-free water, except for a single occurrence of *N*. *stefanellii* in another sulfidic cave [41].

Comparison of body size and ventral channel shape of coexisting extant species from sulfidic caves brought considerable support in favor of competition, and no support at all for ecological filtering (Tables 1 and 2). In the Movile species pair, both the adult body size and the shape of the ventral channel were significantly more different than expected under the null model (body size p < 0.0001, body shape p = 0.002). In the Frasassi species pair, the ventral channel shape differed significantly (p < 0.0001) but the body size did not (p = 0.365). Differences exceeding null model expectations suggest morphological overdispersion that might be due to competitive interactions.

**Table 1:** Difference in body size measured in Euclidean distances for pairs of co-occurring *Niphargus* species and their inferred ancestors.

species pair	Movile	Cave	Frasassi	Cave system
	*Euclidean distance*	*ecological process*	*Euclidean distance*	*ecological process*
extant	6.805	competition (p<0.001)	3.775	random (p = 0.365)
ancestral-lower	7.01	competition (p<0.001)	0.18	filtering (p = 0.023)
ancestral-mean	7.8	competition (p<0.001)	0.25	filtering (p = 0.031)
ancestral-upper	8.59	competition (p<0.001)	0.32	filtering (p = 0.040)

Species pair: upper, mean, lower—COMPARE provides estimates of ancestral states with standard errors. We calculated three estimates of ancestral states, as mean, lower (reconstructed value—standard error) and upper (mean + standard error) and repeated the analyses for all three values.

Ecological process: the putative process leading to either significant similarity or significant difference of coexisting species. The p value indicates the probability that species pair is more similar or different than expected when compared to pairs of species randomly drawn from the corresponding regional species pool.

**Table 2:** Difference in ventral channel measured in Euclidean distances for pairs of co-occurring *Niphargus* species and their inferred ancestors.

species pair	Movile	Cave	Frasassi	Cave system
	*Euclidean distance*	*ecological process*	*Euclidean distance*	*ecological process*
extant	2.8166	competition (p = 0.002)	3.386	competition (p<0.0001)
ancestral-lower	0.9876	random (p = 0.171)	2.63	random (p = 0.07)
ancestral-mean	0.5494	filtering (p = 0.024)	2.69	random (p = 0.056)
ancestral-upper	0.4197	filtering (p = 0.007)	2.704	random (p = 0.055)

Species pair: upper, mean, lower—COMPARE provides estimates of ancestral states with standard errors. We calculated three estimates of ancestral states, as mean, lower (reconstructed value—standard error) and upper (mean + standard error) and repeated the analyses for all three values.

Ecological process: the putative process leading to either significant similarity or significant difference of coexisting species. The p value indicates the probability that species pair is more similar or different than expected when compared to pairs of species randomly drawn from the corresponding regional species pool.

**Fig 3:**
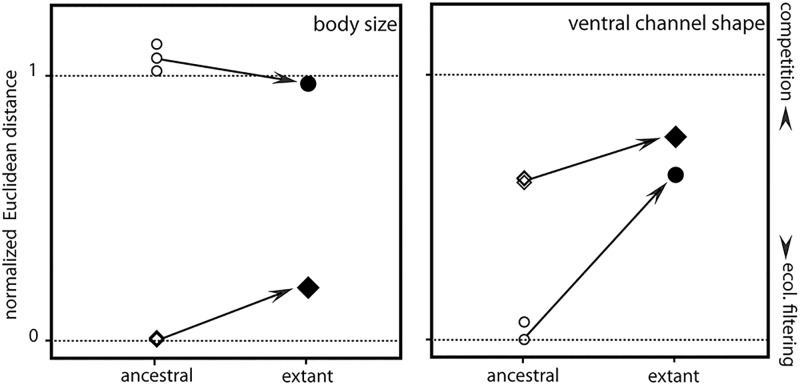
Schematic summary of the results. The y-axis shows the normalized ecological difference between coexisting species as inferred from Euclidean distances, whereas the dashed lines indicate the theoretical minimal and maximal Euclidean distances. Normalized Euclidean distances were calculated as (actual—theoretical minimal Euclidean distance) / (theoretical maximum—theoretical minimal Euclidean distance). Theoretical Euclidean distances were calculated based on regional species pools and can be exceeded in ancestral species pairs. The x axis represents ancestral (open symbols) and extant (solid symbols) species pair. Arrows are oriented from ancestral to extant species pairs for each studied trait. Frasassi species are labeled with diamonds, Movile species with circles. Differences between ancestral species pairs were calculated using the mean, upper and lower estimates for a given trait. Note that the direction of evolutionary change in all but one case indicates competition-driven divergent evolution.

Comparison of extant and ancestral morphologies indicated that evolutionary change occurred in all four species (Tables 1 and 2, Fig 3). In the Movile species pair, we detected substantial divergent change in the shape of the ventral channel. The inferred ancestral ventral channel morphology of both species resembled each other more than expected under the null model (p = 0.024, p value ranging from 0.007 to 0.171 depending on reconstruction, Table 2), while to reach their present-day morphology, it had to diverge by a factor of 2.85–6.71. Body size, on the other hand, differed already significantly more than expected (p < 0.0001) for the reconstructed ancestral species, and the difference in their descendants remained higher than expected (Table 1). Thus, the present-day morphological overdispersion of *Niphargus* body size appears to have originated at the times when Movile Cave was colonized by ancestors of our focal species.

In the Frasassi cave system, we found the direction of evolutionary change to be divergent for both traits. The reconstructed ancestral species were more similar in body size (p = 0.031, p values ranging from 0.023 to 0.040), and marginally more similar in ventral channel shape (p = 0.056, p values ranging from 0.055 to 0.070) than predicted by the null model (Table 2). Euclidean distances, reflecting the respective ecological differences within the ancestral and extant species pair, increased by factor 11.8 to 21.0 times for body size and 1.21 to 1.29 times for ventral channel shape.

## Discussion

We introduce here a new eco-evolutionary study system composed of two pairs of separate, yet closely related species having independently colonized an ecologically extreme environment (sulfidic ground water). The existence of two parallel systems of this kind is globally unique and deserves to be studied. However, the uniqueness of the system at the same time poses methodical limitations to the comparative approach in our study. Inferring ecological processes from current patterns is challenging, in particular when dealing with intrinsic limitations of the model system. Although regional species pools counting four and seven species of a single genus are not small for subterranean environments, testing procedure needed to be adjusted on account of its rigorousness (i.e., using simulated species instead of real ones, see Methods). On the other hand, both cave systems have changed only little over evolutionary time making inference of evolutionary processes potentially more reliable than in other, less stable ecosystems [4]. Also sulfidic concentration has been increased for the past 1–2 and 4–6 million years in Frasassi and Movile [21–22], respectively. Keeping the strengths and omissions of the study in mind, we find results interesting and somewhat unexpected.

Remarkably, amphipod species pairs inhabiting primarily sulfidic environments show no increase in mutual similarity, neither by convergent evolution, nor as compared to random species associations. However, when comparing the ancestral conditions inferred at the time of colonization, similarity in most cases exceeded null model expectations. This shift in patterns from ancestral to recent species pairs possibly reflects a chronology of eco-evolutionary processes during which initial environmental filtering at the time of colonization become superseded by competition-driven divergent evolutionary change [42].

Multiple colonizations of sulfidic habitats have been reported for Mexican cave mollies where researchers found a high degree of morphological and behavioral convergence among independent lineages [18–20]. However, a direct comparison with the Mexican cave molly case is not warranted here as the Mexican sulfidic habitats studied were colonized by a single species only, precluding interspecific competition for resources. In our example, each sulfidic cave was invaded independently by two unrelated niphargid species. Competitive interactions, if they existed at the time of colonization, must have been resolved by divergent evolution of traits associated with resource use. Indeed, we found unexpectedly large morphological differences among coexisting extant species, whereas their inferred ancestral morphologies where very similar. This pattern is in accordance with a competition-driven processes of ecological [43] and community-wide character displacement [44], the latter also called “species sorting” by [43]. However, a simple competition model is not the only possible explanation for the observed pattern. Both body size and the shape of the ventral channel can be subject to multiple selective forces that may yield similar patterns of divergence. Ecological specialization, e.g. shift in habitat use, may not be driven by competition, but can reflect different adaptive peaks [45–46]. In the case of our amphipods, species with slender bodies might prefer small pores, whereas species with stouter bodies might prefer open waters. Furthermore, in Movile Cave predation by a leech and a water bug could have initiated evolutionary divergence in trade-offs between predation and habitat use in our focal *Niphargus* species e.g., [47]. For example, the large body size of *N*. *cf*. *stygius* could make it less exposed to predation e.g., [48], whereas the smaller body size of *P*. *racovitzai* may allow it to position itself upside-down right under the oxygenated upper water layer [21]. Nevertheless, the possibility that the differences observed between our species could be related to differential phylogenetic trends (e.g., Cope's rule; reviewed in [49]) or developmental constraints [49] can be ruled out, as previous studies have shown that small and large body sizes evolve frequently independently [23, 24, 28] and that developmental constraints are unlikely to act on these traits [50].

Whether divergence was driven by competition alone or by a combination with other forces, the two studied functional traits, body size and ventral channel shape, appear not to be subject to long-term convergent selection or to the process of environmental filtering. While evolutionary divergence was expected for body size, we were surprised to observe the same pattern for the ventral channel, since we initially thought that it may play a role in coping with high sulfide concentrations. We can think of two explanations to be tested in future research.

The first one is that most or all *Niphargus* species are able to survive high sulfide concentrations. Two indirect lines of data support this idea. First, *Niphargus* species that live in sulfidic caves belong to different lineages of the genus. The number of sulfide-dwelling lineages might be even higher, as some authors reported other species living in water presenting the characteristic smell of H_2_S [41, 51]. Therefore, it is possible that some form of resistance to sulfide is present in many if not all extant species. In that case, the trait responsible for resistance is likely a plesiomorphic, phylogenetically shared trait [52–53]. Another hint comes from the observations that the Frasassi species pair resist to concentrations of sulfide higher than the ones actually measured in the cave [54]. It is unlikely for selection to produce adaptation exceeding environmental demands. Rather, this high sulfide tolerance might be the side effect of some other physiological mechanism and may therefore represent an exaptation. There is increasing evidence that *Niphargus* species survive anoxic conditions for prolonged periods and that they efficiently recover their oxygen debt [55–57]. Thus, the survival in sulfidic water of various *Niphargus* species may be explained by a general tolerance of this genus to anoxia, possibly coupled with specific behaviors (see below).

The second explanation might be that although sulfide represents a constraint that triggers environmental filtering and convergent selection, we did not look at the traits involved in its detoxification. These may include symbiosis with *Thiotrix* bacteria [58], physiological resistance mechanisms [20, 59–60], or adaptive behaviors that improve oxygen uptake. Plath et al. [18] noted an uptake of atmospheric oxygen in sulfide-dwelling fishes *Poecilla mexicana*. Likewise, regular movements to better oxygenated upper water layers and an upside-down crawling behavior under the water surface were reported in *N*. *ictus* and *P*. *racovitzai*, respectively [21, 26, 58].

In any case, the mechanism of resistance to H_2_S in these species remains unknown. While we were not able to find exclusively shared phenotypic similarities among the two traits studied, it seems unlikely that species inhabiting such extreme environments possess no common adaptations. Future comparative physiological and behavioral research are therefore needed to reveal whether such traits may exist outside the realm of morphology.

## Supporting Information

S1 Table List of species used in the phylogenetic analysis with their localities and the GenBank accession numbers of their sequences. (XLS)

S2 Table Morphometric data used for reconstructing ancestral morphologies. (XLS)

S3 Table Morphological data used for ecological tests. (XLS)

## References

[1] Cornwell WK, Schwilk DW, Ackerly DD. A trait-based test for habitat filtering: convex hull volume. Ecology. 2006; 87: 1465–1471. 16869422 10.1890/0012-9658(2006)87[1465:attfhf]2.0.co;2

[2] Mouchet MA, Burns MDM, Garcia AM, Vieira JP, Mouillot D. Invariant scaling relationship between functional dissimilarity and co-occurrence in fish assemblages of the Patos Lagoon estuary (Brazil): environmental filtering consistently overshadows competitive exclusion. Oikos. 2013; 122: 247–257.

[3] Emerson BC, Gillespie RG. Phylogenetic analysis of community assembly and structure over space and time. Trends Ecol Evol. 2008; 23: 619–630. 10.1016/j.tree.2008.07.005 18823678

[4] Culver DC, Pipan T. The biology of caves and other subterranean habitats. Oxford, UK: Oxford University Press; 2009.

[5] Fargione J, Brown CS, Tilman D. Community assembly and invasion: An experimental test of neutral versus niche processes. Proc Natl Acad Sci USA. 2003; 100: 8916–8920. 12843401 10.1073/pnas.1033107100 PMC166413

[6] Scheffer M, van Nes EH. Self-organized similarity, the evolutionary emergence of similar species. Proc Natl Acad Sci USA. 2006; 103: 6230–6235. 16585519 10.1073/pnas.0508024103 PMC1458860

[7] Chesson P. Mechanisms of maintenance of species diversity. Annu Rev Ecol Syst. 2000; 31: 343–366.

[8] Stubbs WJ, Wilson JB. Evidence for a limiting similarity in a sand dune community. J Ecol. 2004; 92: 557–567.

[9] Wilson JB, Stubbs WJ. Evidence for assembly rules: limiting similarity within a saltmarsh. J Ecol. 2011; 100: 210–221.

[10] Vergnon R, Leijs R, van Nes EH, Scheffer M. Repeated parallel evolution reveals limiting similarity in subterranean diving beetles. Am Nat. 2013; 182: 67–75. 10.1086/670589 23778227

[11] Kraft NJB, Valencia R, Ackerly DD. Functional traits and niche-based tree community assembly in an Amazonian forest. Science. 2008; 322: 580–582. 10.1126/science.1160662 18948539

[12] Ingram T, Shurin JB. Trait-based assembly and phylogenetic structure in northeast Pacific rockfish assemblages. Ecology. 2009; 90: 2444–2453. 19769123 10.1890/08-1841.1

[13] Schluter D. The Ecology of Adaptive Radiation. Oxford: Oxford University Press; 2000.

[14] Moen DS, Smith SA, Wiens JJ. Community assembly through evolutionary diversification and dispersal in Middle American treefrogs. Evolution. 2009; 63: 3228–3247. 10.1111/j.1558-5646.2009.00810.x 19663988

[15] Fišer C. *Niphargus*: A model system for evolution and ecology In: Culver DC, White WB editors. Encyclopedia of Caves. Academic Press; 2012 pp. 555–564.

[16] Nicholls P. The effect of sulphide on cytochrome *aa3* isosteric and allosteric shifts of the reduced α-peak. Biochim Biophys Acta. 1975; 396: 24–35. 807256 10.1016/0005-2728(75)90186-3

[17] Tobler M, Schlupp I, Heubel K, Riesch R, de Leon FJG, Giere O, et al Life on the edge: hydrogen sulfide and the fish communities of a Mexican cave and surrounding waters. Extremophiles. 2006; 10: 577–585. 16788733 10.1007/s00792-006-0531-2

[18] Plath M, Tobler M, Riesch R, de León FJG, Giere O, Schlupp I. Survival in an extreme habitat: the roles of behaviour and energy limitation. Naturwissenschaften. 2007; 94: 991–996. 17639290 10.1007/s00114-007-0279-2

[19] Tobler M, DeWitt TJ, Schlupp I, de Leon FJG, Herrmann R, Feulner P, et al Toxic hydrogen sulfide and dark caves: phenotypic and genetic divergence across two abiotic environmental gradients in *Poecilia mexicana* . Evolution. 2008; 62: 2643–2649. 10.1111/j.1558-5646.2008.00466.x 18637957

[20] Tobler M, Palacios M, Chapman LJ, Mitrofanov I, Bierbach D, Plath M, et al Evolution in extreme environments: replicated phenotypic differentiation in livebearing fish inhabiting sulfidic springs. Evolution. 2011; 65: 2213–2228. 10.1111/j.1558-5646.2011.01298.x 21790570

[21] Sarbu SM. Movile Cave: a chemoautotrophically based groundwater ecosystem In Wilkens H, Culver DC, Humphreys WF editors. Subterranean ecosystems. Amsterdam: Elsevier; 2000 pp. 319–343.

[22] Sarbu SM, Galdenzi S, Menichetti M, Gentile G. Geology and biology of the Frasassi caves in central Italy: an ecological multi-disciplinary study of a hypogenic underground karst system In Wilkens H, Culver DC, Humphreys WF editors. Subterranean ecosystems. Amsterdam: Elsevier; 2000 pp. 359–378.

[23] Trontelj P, Blejec A, Fišer C. Ecomorphological convergence of cave communities. Evolution. 2012; 66: 3852–3865. 10.1111/j.1558-5646.2012.01734.x 23206142

[24] Fišer C, Zagmajster M, Zakšek V. Coevolution of life history traits and morphology in female subterranean amphipods. Oikos. 2013; 122: 770–778.

[25] Flot JF, Bauermeister J, Brad T, Hillebrand-Voiculescu A, Sarbu SM, Dattagupta S. *Niphargus*–*Thiothrix* associations may be widespread in sulphidic groundwater ecosystems: evidence from southeastern Romania. Mol Ecol. 2013; 23: 1405–1417. 10.1111/mec.12461 24044653 PMC4282457

[26] Flot JF, Wörheide G, Dattagupta S. Unsuspected diversity of *Niphargus* amphipods in the chemoautotrophic cave ecosystem of Frasassi, central Italy. BMC Evol Biol. 2010; 10: 171 10.1186/1471-2148-10-171 20534131 PMC2896373

[27] Ruffo S, Stoch F. Checklist e distribuzione della fauna italiana. 10.000 Specie Terrestri E Delle Acque Interne. Memoire del Museo Civico di Storia Naturale di Verona, 2. serie, Sezione Scienze della Vita 16; 2005.

[28] Fišer C, Sket B, Trontelj P. A phylogenetic perspective on 160 years of troubled taxonomy of *Niphargus* (Crustacea: Amphipoda). Zool Scr. 2008; 37: 665–680.

[29] Trontelj P, Douady CJ, Fišer C, Gibert J, Gorički Š, Lefebure T, et al A molecular test for cryptic diversity in ground water: how large are the ranges of macrostygobionts? Freshwater Biol. 2009; 54: 727–744.

[30] Katoh K, Kuma K, Toh H, Miyata T. MAFFT version 5: improvement in accuracy of multiple sequence alignment. Nucleic Acids Res. 2005; 33: 511–518. 15661851 10.1093/nar/gki198 PMC548345

[31] Talavera G, Castresana J. Improvement of phylogenies after removing divergent and ambiguously aligned blocks from protein sequence alignments. Syst Biol. 2007; 56: 564–577. 17654362 10.1080/10635150701472164

[32] Ronquist F, Huelsenbeck JP. MrBayes 3: Bayesian phylogenetic inference under mixed models. Bioinformatics. 2003; 19: 1572–1574. 12912839 10.1093/bioinformatics/btg180

[33] Fišer C, Trontelj P, Luštrik R, Sket B. Toward a unified taxonomy of *Niphargus* (Crustacea: Amphipoda): a review of morphological variability. Zootaxa. 2009; 2061: 1–22.

[34] Dahl E. The amphipod functional model and its bearing upon systematics and phylogeny. Zool Scr. 1977; 6: 221–228.

[35] Gotelli NJ, Graves RG. Null models in ecology. Washington and London: Smithsonian Institution Press; 1996.

[36] Rabosky DL, Reid J, Cowan MA, Foulkes J. Community-wide overdispersion of body size in Australian desert lizard communities. Oecologia. 2007; 154: 561–570. 17874134 10.1007/s00442-007-0849-1

[37] RDevelopmentCoreTeam. R: a language and environment for statistical computing R Foundation for Statistical Computing, Vienna, Austria 2014 Available: http://www.R-project.org.

[38] Schluter D, Price T, Mooers AO, Ludwig D. Likelihood of ancestor states in adaptive radiation. Evolution. 1997; 51: 1699–1711. 28565128 10.1111/j.1558-5646.1997.tb05095.x

[39] Butler AM, King AA. Phylogenetic comparative analysis: a modeling approach for adaptive evolution. Am Nat. 2004; 164: 683–695. 29641928 10.1086/426002

[40] Martins EP. COMPARE, version 4.6b. Computer programs for the statistical analysis of comparative data. Department of Biology, Indiana University, Bloomington IN. 2004. Available: http://compare.bio.indiana.edu/.

[41] Latella L, Di Russo C, De Pasquale L, Dell’Anna L, Nardi G, Rampini M. Preliminary investigations of a new sulfurous cave in central Italy. Mém Biospéol. 1999; 26: 131–135.

[42] Weiher E, Freund D, Bunton T, Stefanski A, Lee T, Bentivenga S. Advances, challenges and a developing synthesis of ecological community assembly theory. Phil. Trans. R. Soc. B. 2011; 366 10.1098/rstb.2011.0056 PMC3130429 21768155

[43] Stuart YE, Losos JB. Ecological character displacement: glass half full or half empty? Trends Ecol Evol. 2013; 28: 402–408. 10.1016/j.tree.2013.02.014 23537690

[44] Dayan T, Simberloff D. Ecological and community wide character displacement. Ecol Lett. 2005; 8: 875–894.

[45] Losos JB. Community evolution in Greater Antillean Anolis lizards: phylogenetic patterns and experimental tests. Philos T Roy Soc B. 1995; 349: 69–75.

[46] Martin RA, Pfennig DW. Disruptive selection in natural populations: the roles of ecological specialization and resource competition. Am Nat. 2009; 174: 268–281. 10.1086/600090 19527118

[47] Cothran RD, Henderson KA, Schmindenberg D, Relyea R. Phenotypically similar but ecologically distinct: differences in competitive abilities and predation risk among amphipods. Oikos. 2013; 122: 1429–1440.

[48] Olson SL, Hearty PJ. Predation as the primary selective force in recurrent evolution of gigantism in *Poecilozonites* land snails in Quaternary Bermuda. Biol Lett. 2010; 6: 807–810. 10.1098/rsbl.2010.0423 20554560 PMC3001380

[49] Blanckenhorn WU. The evolution of body size: what keeps organisms small? Q Rev Biol. 2000; 75: 385–407. 11125698 10.1086/393620

[50] Fišer C, Bininda-Emonds ORP, Blejec A, Sket B. Can heterochrony explain the high morphological diversity within the genus *Niphargus* (Crustacea: Amphipoda)? Org Divers Evol. 2008; 8: 146–162.

[51] Sket B. Distribution, ecological character, and phylogenetic importance of *Niphargus valachicus* . Biol Vest. 1981; 29: 87–103.

[52] Wiens JJ. Commentary on Losos (2008): niche conservatism déjà vu. Ecol Lett. 2008; 11: 1004–1005. 10.1111/j.1461-0248.2008.01238.x 18808496

[53] Best RJ, Caulk NC, Stachowicz JJ. Trait vs. phylogenetic diversity as predictors of competition and community composition in herbivorous marine amphipods. Ecol Lett. 2013; 16: 72–80. 10.1111/ele.12062 23066869

[54] Bauermeister J, Assig K, Dattagupta S. Exploring the sulfide tolerance of ectosymbiotic *Niphargus* amphipods from the Frasassi caves, central Italy. Int J Speleol. 2013; 42: 141–145.

[55] Hervant F, Mathieu J, Messana G. Oxygen consumption and ventilation in declining oxygen tension and posthypoxic recovery in epigean and hypogean crustaceans. J Crust Biol. 1998; 18: 717–727.

[56] Malard F, Hervant F. Responses to low oxygen In: Culver DC, White WB editors. Encyclopedia of Caves. Academic Press; 2012 pp. 651–658

[57] Lawniczak M, Romestaing C, Roussel D, Maazouzi C, Renault D, Hervant F. Preventive antioxidant responses to extreme oxygen level fluctuation in a subterranean crustacean. Comp Biochem Phys A. 2013; 165: 299–303. 10.1016/j.cbpa.2013.03.028 23545443

[58] Dattagupta S, Schaperdoth I, Montanari A, Mariani S, Kita N, Valley JW, et al A novel symbiosis between chemoautotrophic bacteria and a freshwater cave amphipod. The ISME Journal. 2009; 3: 935–943. 10.1038/ismej.2009.34 19360027

[59] Tobler M, Henpita C, Basset B, Kelly JL, Shaw JH. H_2_S exposure elicits differential expression of candidate genes in fish adapted to sulfidic and non-sulfidic environments. Comparative biochemistry and physiology. Comp Biochem Phys A. 2014;174: 7–14. 10.1016/j.cbpa.2014.04.012 24813672

[60] Pfenninger M, Lerp H, Tobler M, Passow C, Kelley JL, Funke E, et al Parallel evolution of *cox* gene in H_2_S tolerant fish as key adaptation to a toxic environment. Nature Communications. 2014; 5: 3873 10.1038/ncomms4873 24815812

[61] Zagmajster M, Eme D, Fišer C, Galassi D, Marmornier P, Stoch F, et al Geographic variation in range size and beta diversity of groundwater crustacean: insights from habitats with low thermal seasonality. Global Ecol Biogeogr. 2014; 23: 1135–1145.

